# Supplementary material for: Locally coupled electromechanical interfaces based on cytoadhesion-inspired hybrids to identify muscular excitation-contraction signatures
Source: Nat Commun. 2020 May 4;11:2183. doi: 10.1038/s41467-020-15990-7 (PMC7198512; doi:10.1038/s41467-020-15990-7)
Supplement: Supplementary file 1 — Supplementary Information [file 41467_2020_15990_MOESM1_ESM.pdf]

Supplementary Information

Locally coupled electromechanical interfaces based on cytoadhesion-inspired hybrids to identify muscular excitation-contraction signatures

Pingqiang Cai[1] *et al.*

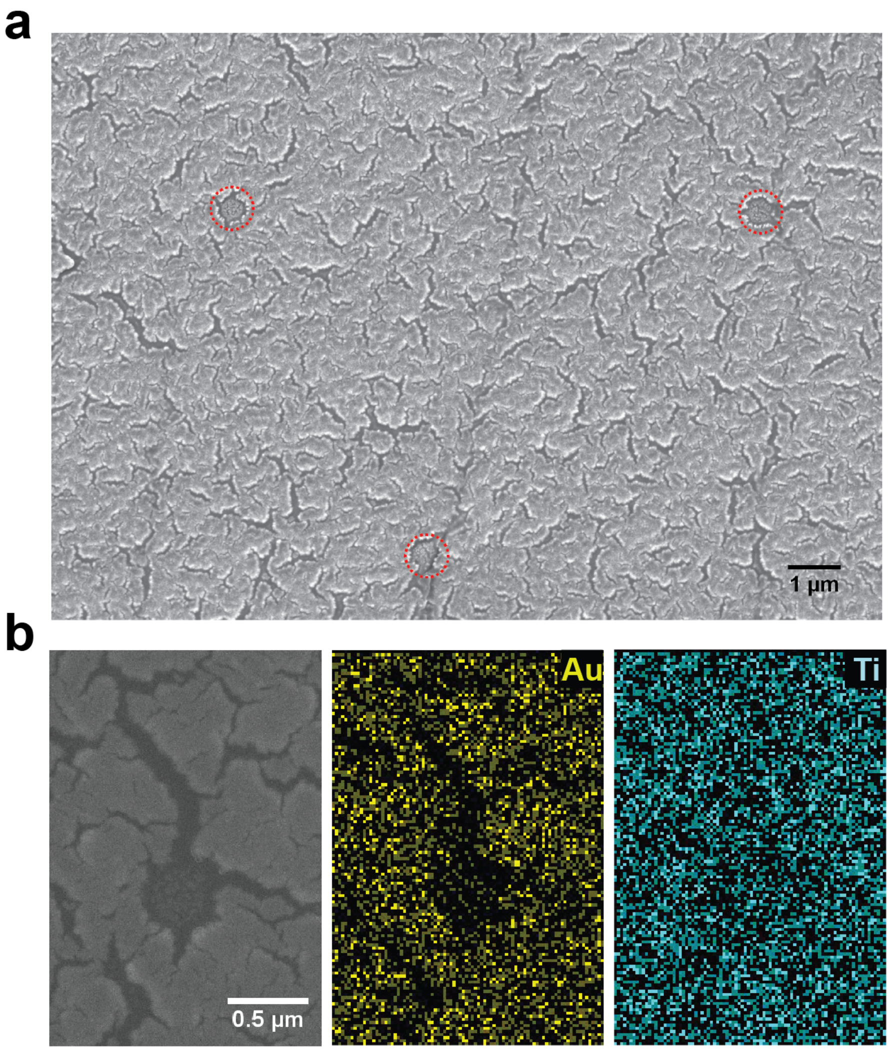

Supplementary Figure 1 | Structural characterization and elemental analysis metallic nanofilm a, SEM image of Au nanofilm@elastomer shows that "black holes" with smashed gold speckles were dispersed among the tri-branched microcracks. **b,** SEM image and EDX mapping show the homogeneous distribution of Ti over the film, in contrast to the absence of Au at microcracks. Representative of two samples.

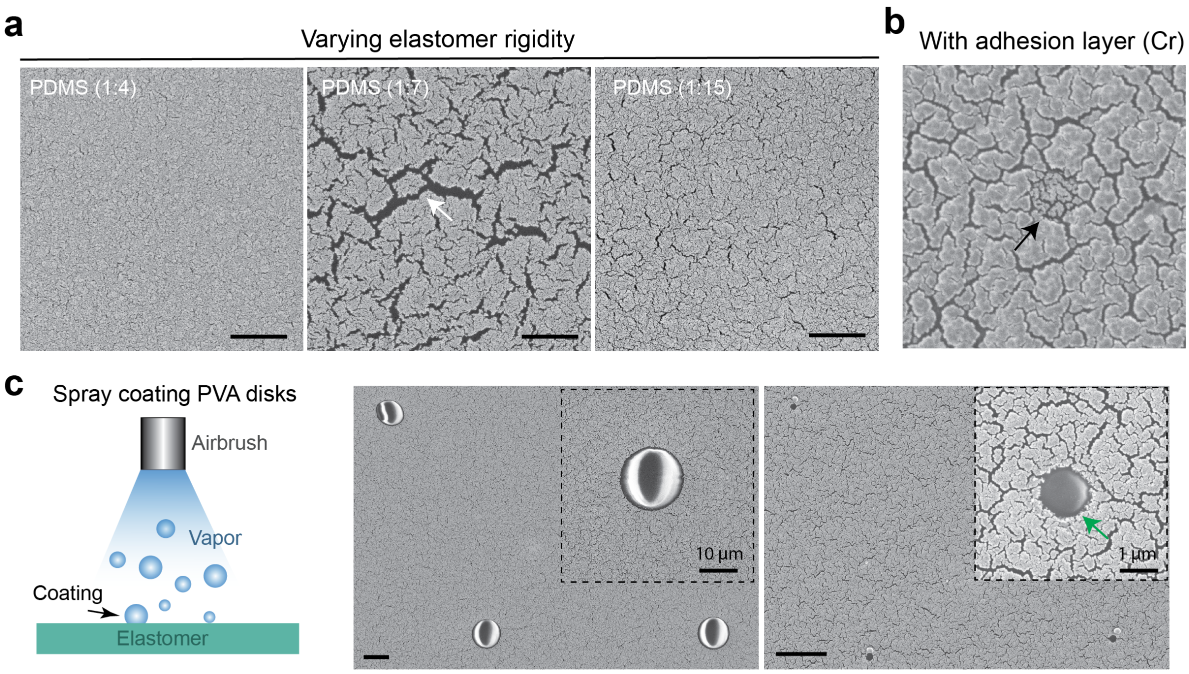

Supplementary Figure 2 | Modulation of the microstructure of metallic nanofilms. SEM images show the influence of **a**) substrate rigidity and **b**) adhesion on forming microcracked Au nanofilm. The variability in substrate rigidity influenced the size and distribution of microcracks, as well as the formation of "smashed Au speckles". The increase of substrates rigidity seemed to promote the adhesion but reduce the mobility of Au NPs, as evident from the relatively continuous Au film on PDMS (1:4) substrates. On PDMS (1:7) substrates, the "hole-like" structure presented small gold patches (*White arrow*) in the centre and several long cracks at the periphery. This suggests that the formation of "hole-like" structures might be initiated by the convergence of different propagating microcracks. *Black arrow* in (b) indicates that the promoted adhesion (with 3 nm Cr) can impede the formation of "smashed gold particles" in the "hole". It suggests that conditions can be stringent for forming the "hole-like" structure with smashed Au speckles, probably demanding the equilibrium between AuNPs mobility and adhesion, as well as the equilibrium between energy storage and release[1]. Scale bar: 1 μm. **c,** Schematic and SEM images show that the size of "hole-like" microstructures can be modulated when spray-coating PVA disks as the sacrifice masks. Interestingly, the "holes" (*Green arrow*) were free of gold deposition, which might be beneficial for the penetration of tough gel, therefore the formation of "adhesion plaques". Scale bar: 10 μm.

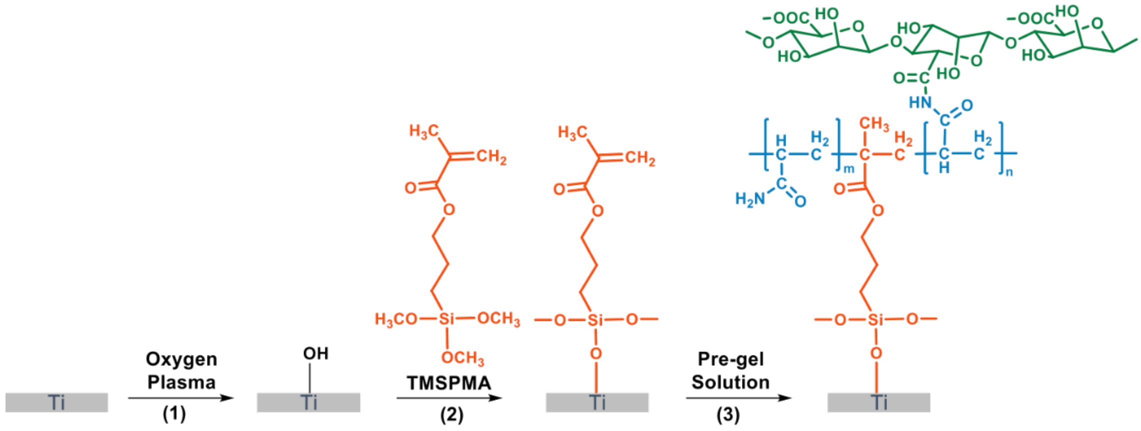

Supplementary Figure 3 | Schematic shows the procedure to achieve interfacial tough bonding.

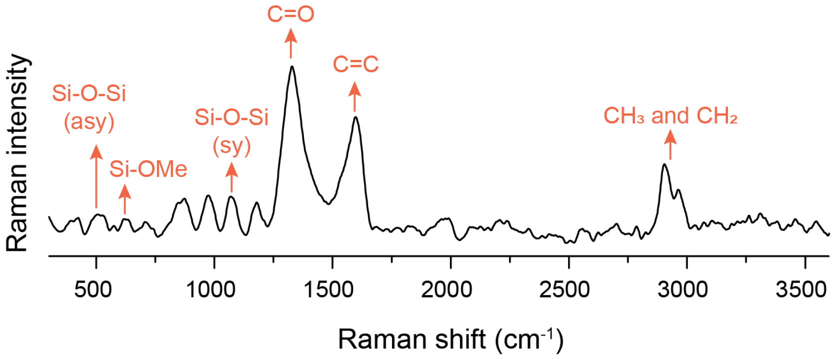

Supplementary Figure 4 | Raman spectra of TMSPMA-treated metallic nanofilm on the elastomer. The spectrum exhibits the characteristic absorption peaks at 1327 cm^{-1} (C=O band), 1598 cm^{-1} (C=C band), 2903 cm^{-1} (CH_3 and CH_2 stretching band), indicating the successful binding of TMSPMA molecules. The condensation reactions are indicated by the absorption peaks at 498 and 1070 cm^{-1} (Si-O-Si linkages), and the low intensity of absorption peak at 619 cm^{-1} (Si-OMe band).

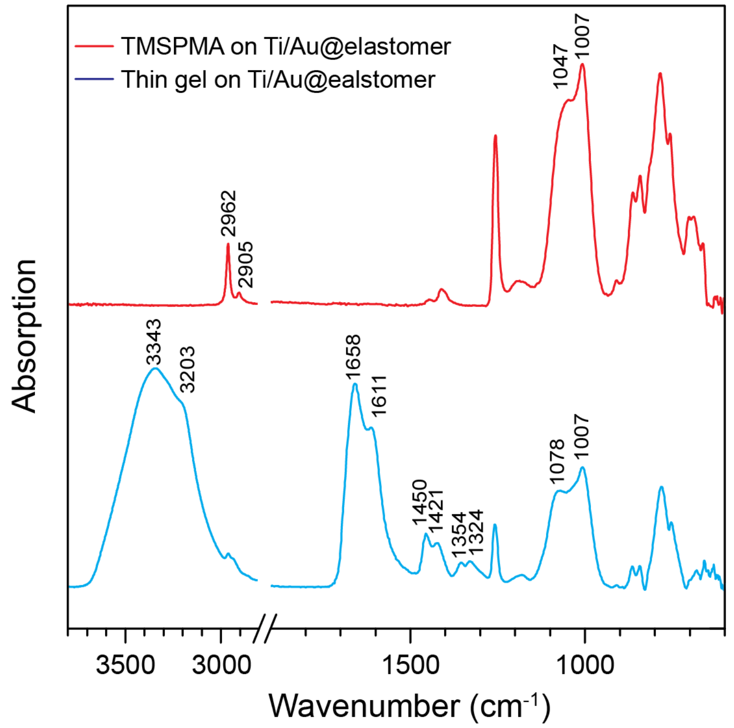

Supplementary Figure 5 | FTIR spectra of *TMSPMA-treated Ti/Au@elastomer* before and after gel casting. It indicates the successful grafting of TMSPMA and subsequent anchoring of tough gels. In the spectra of *TMSPMA on Ti/Au@elastomer*, the grafting of TMSPMA onto Ti surfaces is characterized by the absorption band at 1007 cm^{-1} arising from Si-O-Ti bridges, the band at 1047 cm^{-1} due to the stretching vibration of Si-O bonds, and the band at 2962 and 2905 cm^{-1} attributed to the stretching vibration of –CH_2 groups. The characteristic absorption band at 1719 cm^{-1} (stretching vibration of –CO- group) and at 1636 cm^{-1} (vinyl group) is not detected in the as-described condition. The spectra of *Thin gel on Ti/Au@elastomer* shows polyacrylamide with the characteristic absorption band of 3343 cm^{-1} and 3203 cm^{-1} corresponding to the stretching vibration of N-H, the band at 1611 cm^{-1} for primary amine (N-H deformation), 1450 cm^{-1} for CH_2 in-plane scissoring, 1421 for cm^{-1} for primary amine (C-N stretching), 1354 cm^{-1} for C-H deformation. The absorption at 1324 cm^{-1} for C-H deformation with secondary alcohols indicates the network of alginate gel. The formation of the Si-O-Ti bridge is shown by the absorption band at 1007 cm^{-1}, and the stretching vibration of Si-O bonds band shifted to 1078 cm^{-1}, probably caused by the C-O stretching in secondary alcohols.

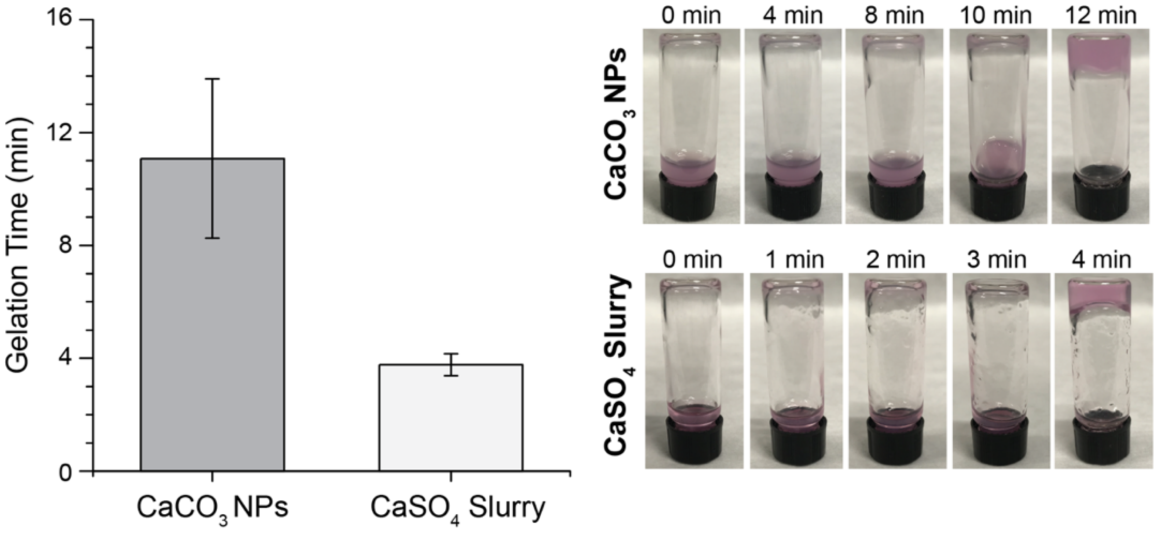

Supplementary Figure 6 | Prolonged gelation using $CaCO_3$ nanopowder as a reservoir of Ca^{2+} crosslinkers. 0.01% phenol red was added. Data are presented as mean ± s.d.

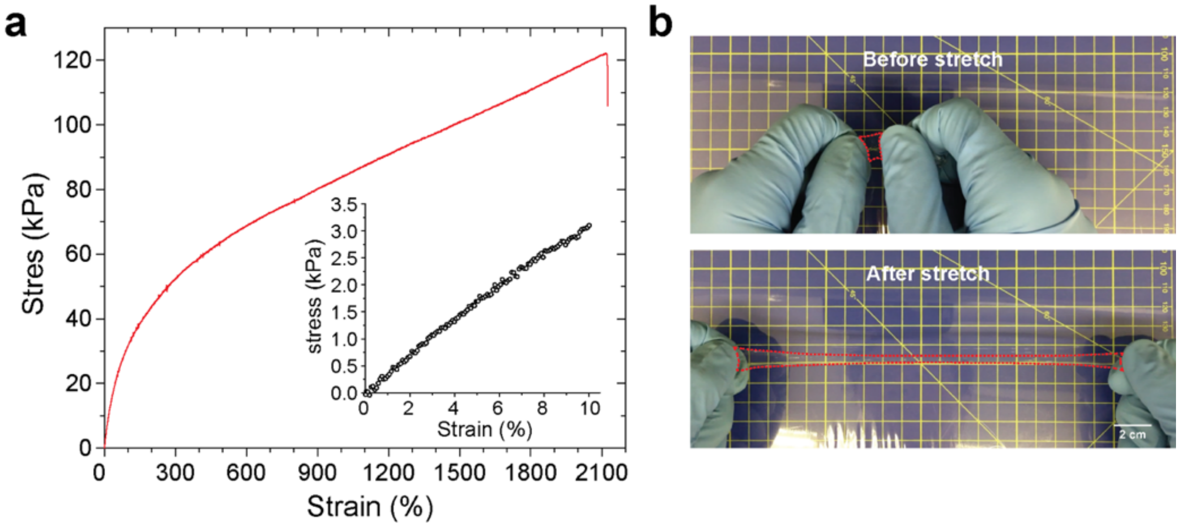

Supplementary Figure 7 | Mechanical characterization of the tough gel. a, Plots show the stretchability of the as-prepared tough hydrogel, comprising of the chemical network of polyacrylamide and the physical network alginate/Ca^{2+}. The maximum strain was up to ~2130 %. The inset graph shows Young's modulus of the tough gel was estimated as 31 kPa. **b,** Photographs show the tough gel could be manually stretched to about 20 times of its original length.

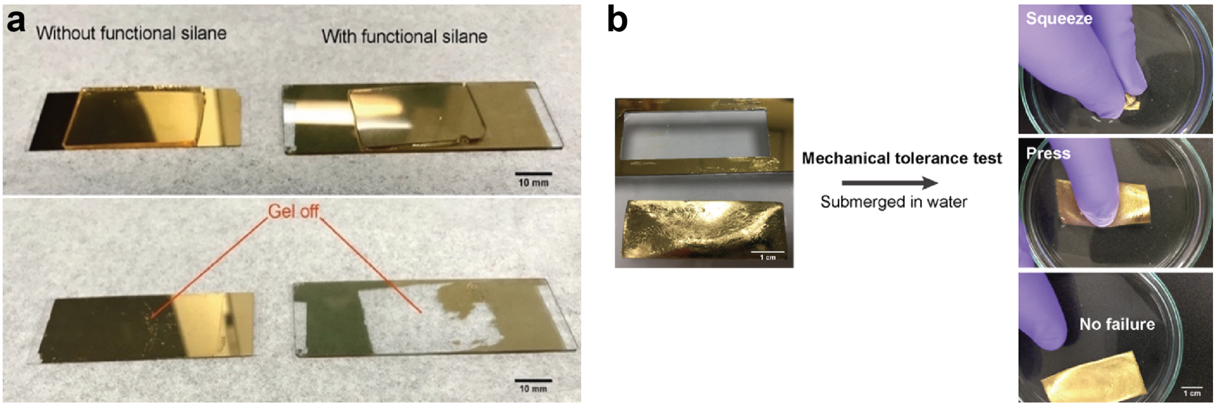

Supplementary Figure 8 | Demonstration of tough interfacial bonding formation. a, Photograph demonstrates the successful bonding of tough gel to the double metallic nanofilm with functionalized saline. **b,** Photographs show the mechanical tolerance of bonding between the gel and the double metallic nanofilm.

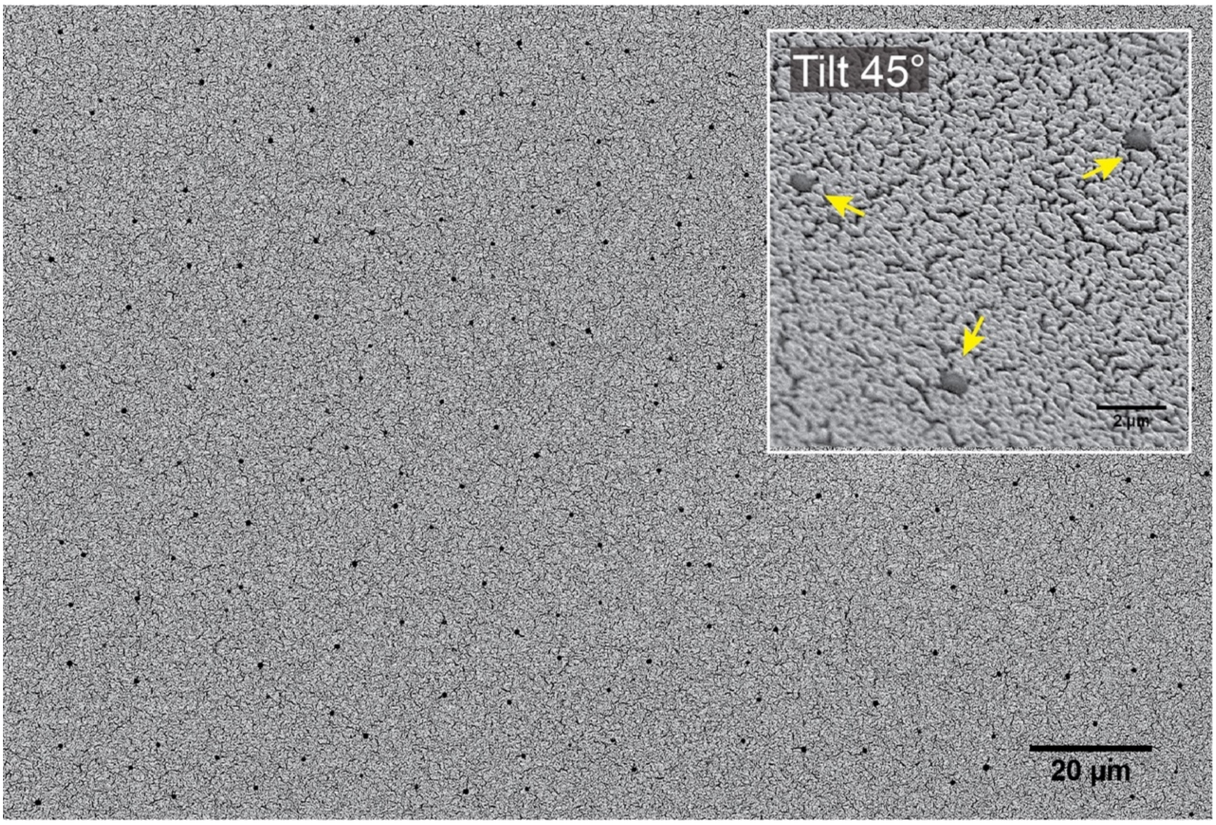

Supplementary Figure 9 | Even dispersion of "adhesion plaques" over a large area. SEM images show the evenly distributed "adhesion plaques" over a large area that sprouted out of the metallic nanofilm (*Inset*), after the elastomer layer of *CoupOn* was removed.

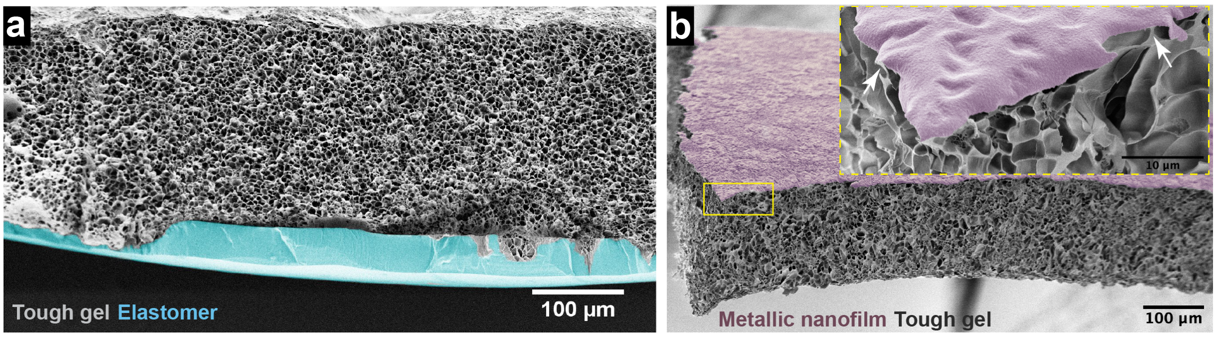

Supplementary Figure 10 | Characterization of the multilayer structure. a, Cross-sectional SEM image shows the consistent thickness of tough gel (*grey color*) and elastomer layer (*cyan color*) of the fabricated *CoupOn*. Upon flash freezing and cryo-cutting in liquid nitrogen, the samples were submerged in water and liquid nitrogen again to minimize the drying-caused delamination, though gel swelling was incurred. Representative of four samples. **b,** Cross-sectional SEM image of the *CoupOn hybrid* with the elastomer removed. *White arrows* in the inset indicate the intimate contact between the tough gel (*grey color*) and the metallic nanofilm (*magenta color*), implying the formation of the chemical bond at their interface. Representative of four samples.

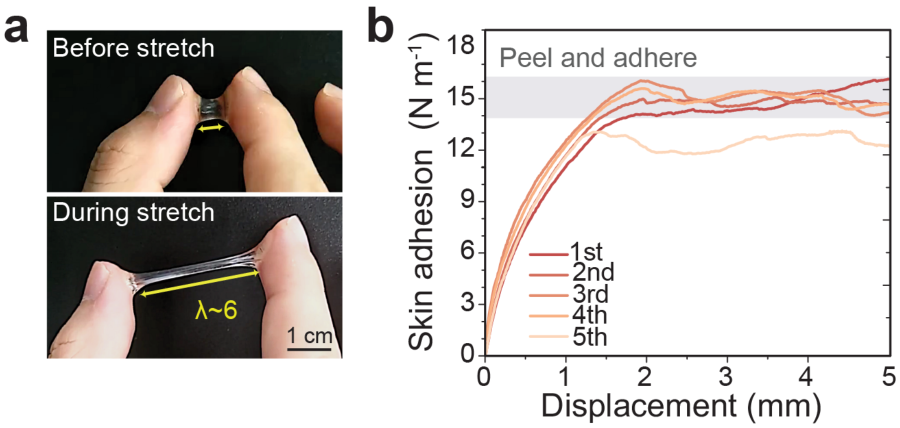

Supplementary Figure 11 | On-skin adhesion and stretchability of the tough gel. a, Photographs show the ionic gel with uncompromised adhesion to the skin when subjected to ~ 500 % strain. **b,** Plot shows repeated adhesion of *CoupOn* on porcine skin.

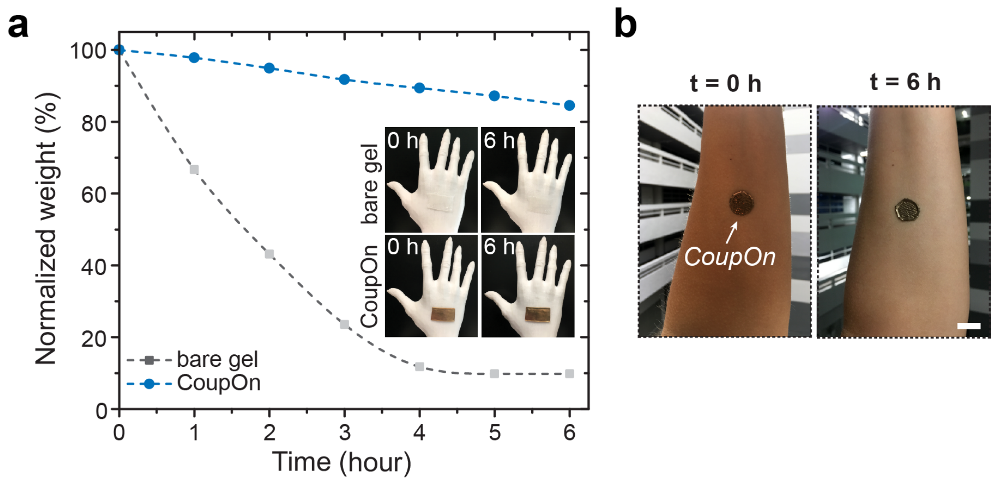

Supplementary Figure 12 | Dehydration mitigation enabled by the bonded elastomer. a, Plot shows the weight loss of the *CoupOn* hybrid and bare gel after 6-hour exposure to the ambient environment. **b,** Photographs show that the mitigated dehydration allowed 6-hour wearing of the CoupOn hybrid without drying or delamination.

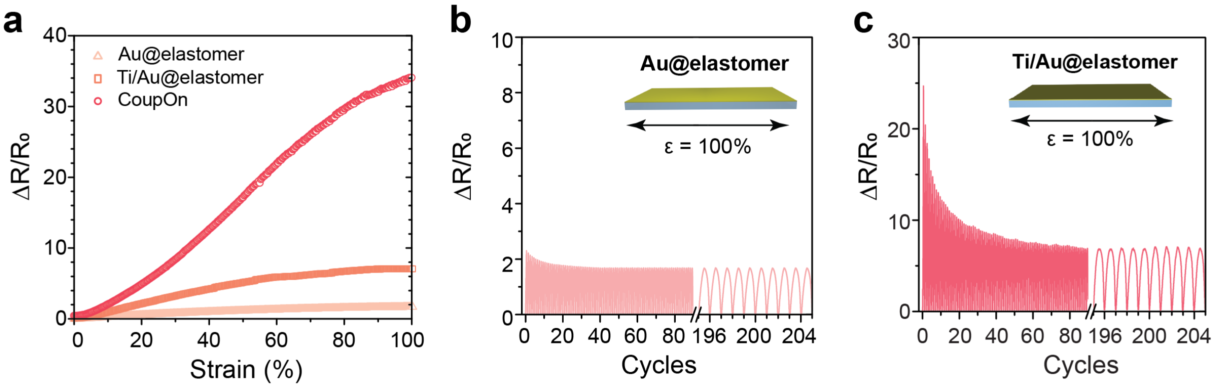

Supplementary Figure 13 | Increase of gauge factor by introducing Ti and tough gel. a, Circle plot shows the relative resistivity change of Au@elastomer, Au/Ti@elastomer and *CoupOn* with regards to subjected strain, respectively. Plots show the respective resistivity change of (**b**) Au@elastomer and (**c**) Ti/Au@elastomer bearing 100% strain over 200 cycles.

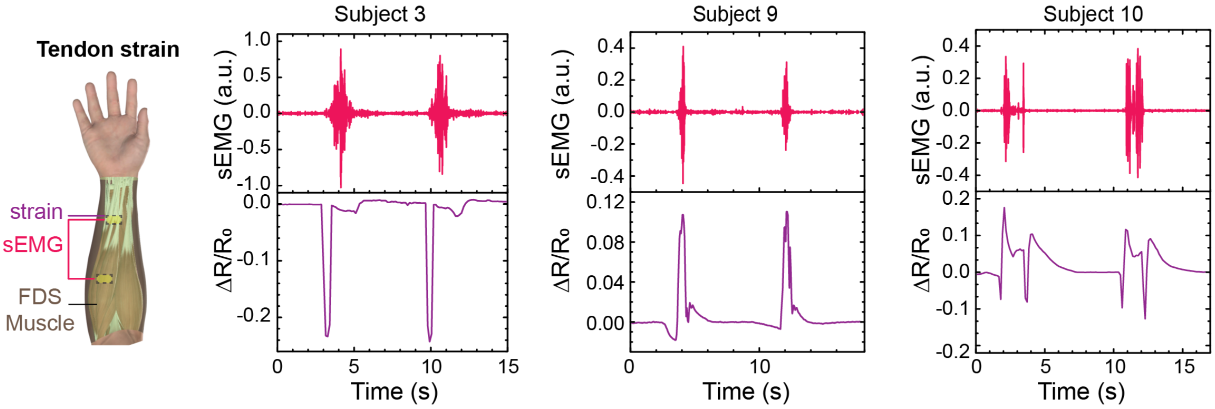

Supplementary Figure 14 | Distinctive excitation-contraction signatures during the fast 20 kg grips. sEMG of subject 9 and 10 are normalized with respective sEMG signals in fast grips at the 30 kg force level.

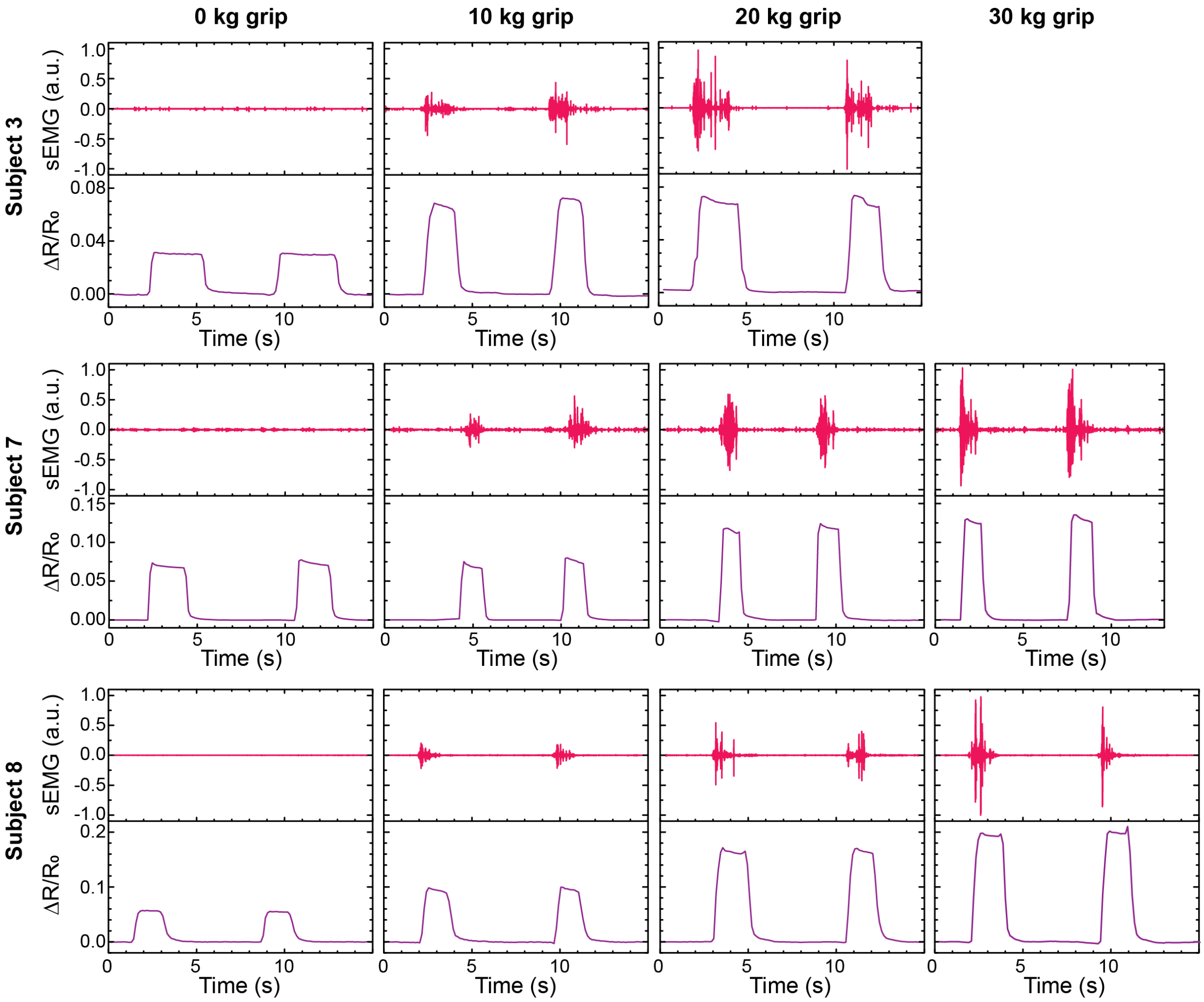

Supplementary Figure 15 | Graphs show the muscular excitation-contraction signatures. Representative of 2-4 sets of each gesture at each force level. EMG of subject 9 and 10 are normalized with respective sEMG signals in fast grips at the 30 kg force level.

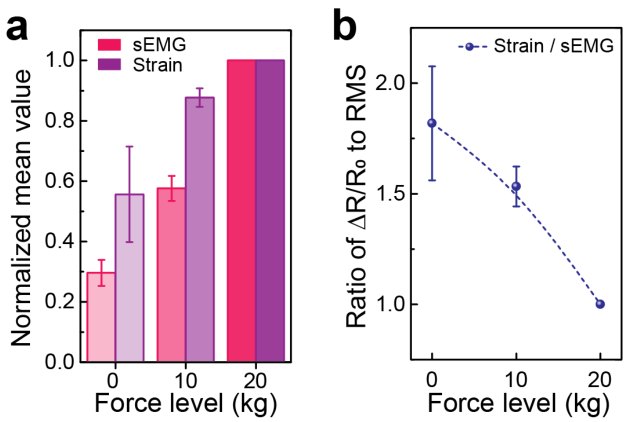

Supplementary Figure 16 | Electromechanical coupling analysis of subject 1-3. a, Bar chart shows the mean normalized level of sEMG and strain signals. **b,** Plot shows the ratio of normalized mean normalized level of strain to sEMG signals. Data are normalized using respective signal amplitudes at 20 kg grip forces of subject 1-3 and presented as mean ± s.e.m.

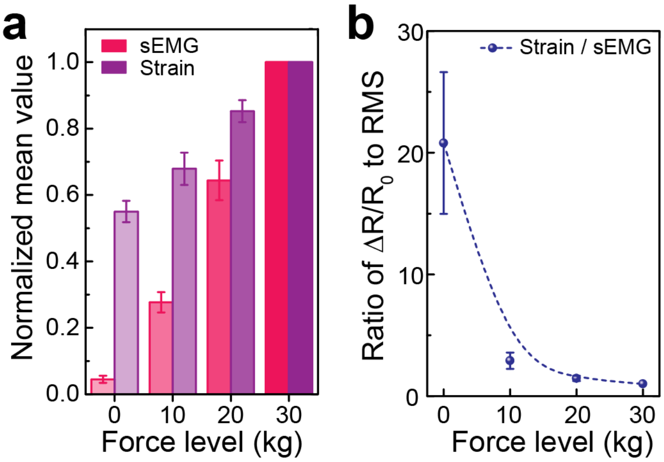

Supplementary Figure 17 | Electromechanical coupling analysis of subject 4-11. a, Bar chart shows the mean value of sEMG and strain signals. **b,** Plot shows the ratio of normalized mean value of strain to sEMG signals. Data are normalized using respective signal amplitudes at 30 kg grip forces of subject 4-11 and presented as mean ± s.e.m.

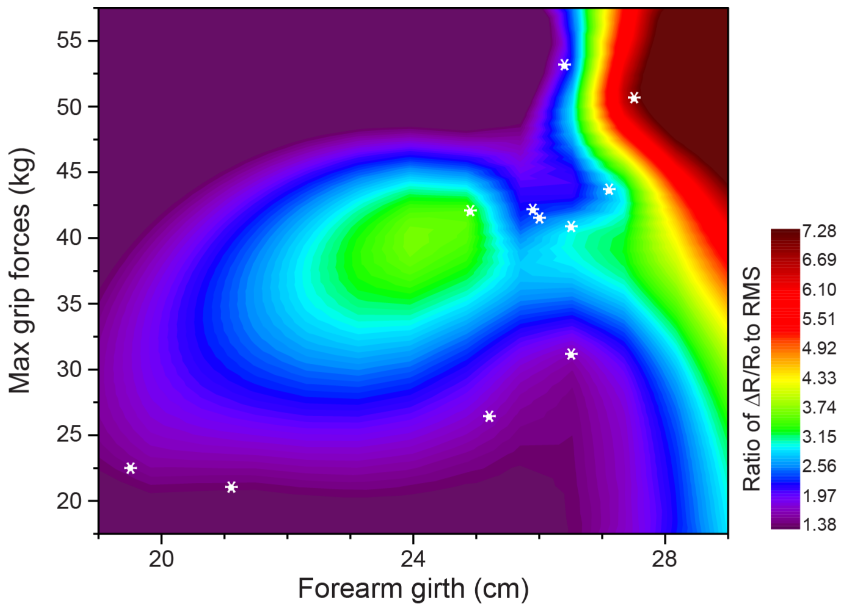

Supplementary Figure 18 | Diagram shows the correlation of the electromechanical coupling efficiency with subjects' forearm girth and max grip forces. Data obtained from the 11 subjects during 10 kg grips. sEMG of subject 1-3 and subject 4-11 are respectively normalized against 20 kg grips and 30 kg grips. *White asterisks* locate each subject by the max grip force and forearm girth. Color coding and fitting conducted in Origin 9.1.

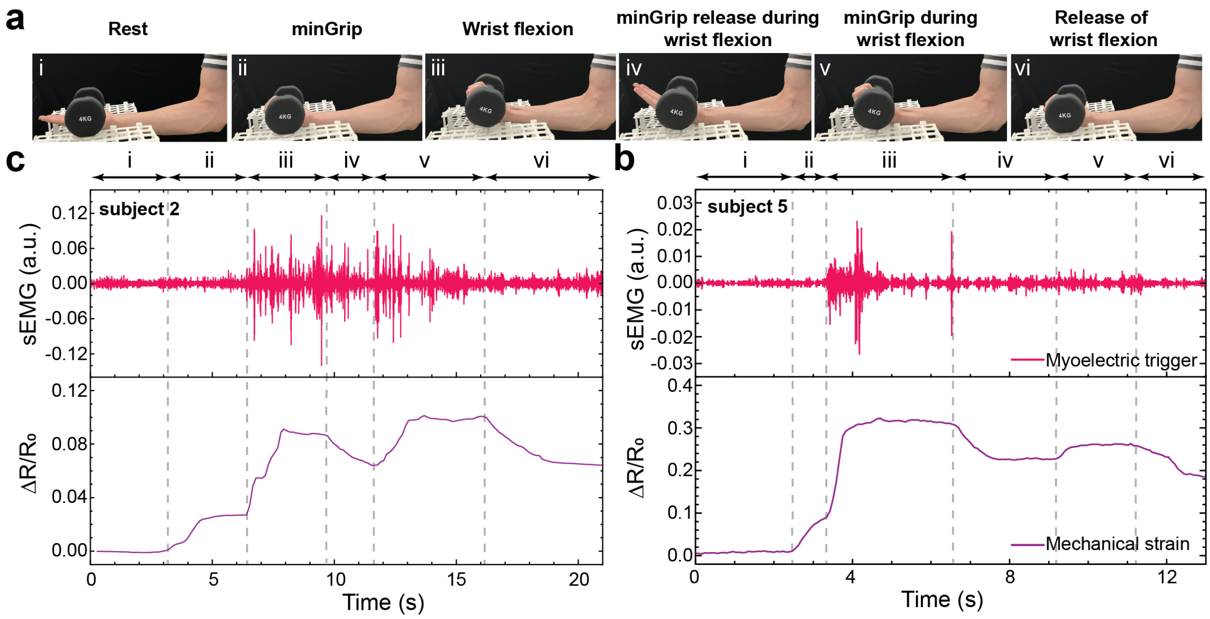

Supplementary Figure 19 | Resolving low-force and high-force components in the "resist and grip" gesture. a, Photographs show the 6 steps in the "resist and grip" gesture. The elbow angle kept constant during the whole process. The dumbbell was 4 kg, with the rod diameter of 4.3 cm. **b-c,** Plots show the excitation-contraction signatures of subject 5 and 2 when performing the "resist and grip" gesture. Patterns of the mechanical strain of the two subjects appear similar, though their sEMG signals appear distinct. Low-force component can be resolved from the high-force gestures through analyzing the electromechanical coupling. For subject 5, minGrip release (step iv) and minGrip (step v) while maintaining the wrist flexion exhibit little distinguishable characteristics in the sEMG signal, but obvious two plateau in the mechanical strain signal. sEMG signals of subject 2 and 5 are respectively normalized with their respective signals during the standard 20 kg grip and 30 kg grip.

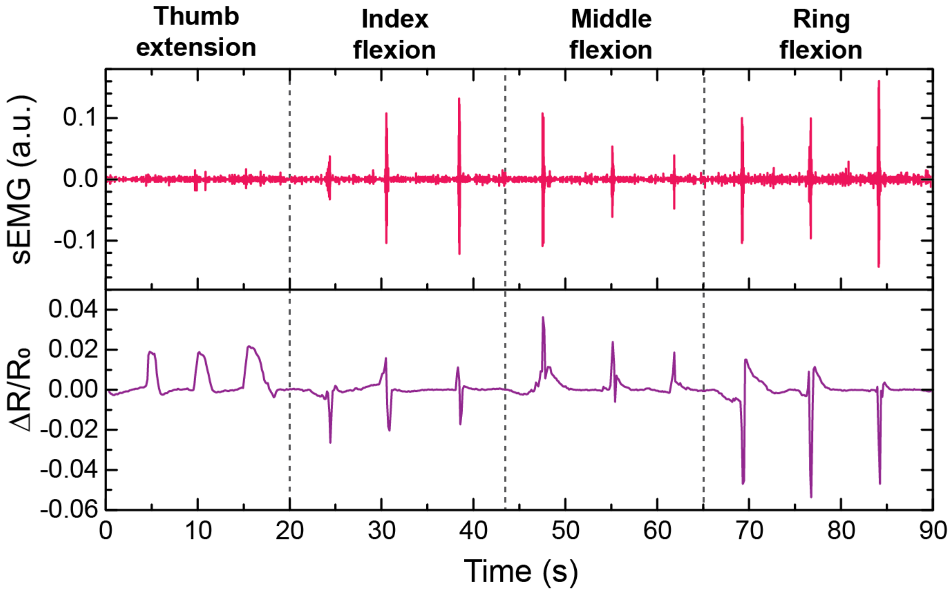

Supplementary Figure 20 | Distinct excitation-contraction signatures of different finger gestures. Data obtained from subject 2, using a finger exerciser and the tendon strain-sensing *CoupOn* placed above the origin of FDS muscle. sEMG signals are normalized with the signals during standard 20 kg grips.

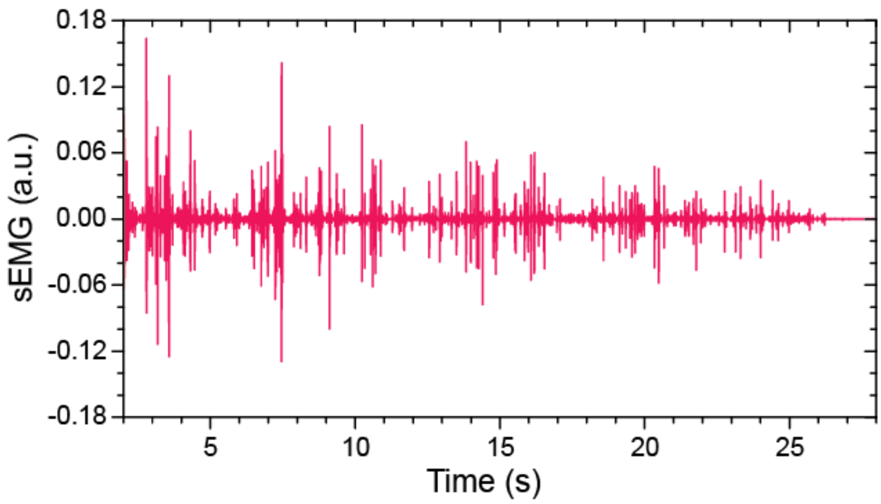

Supplementary Figure 21 | Decaying sEMG peak amplitude during muscle fatigue. Plot shows the data range cropped from Fig. 5f and rescaled.

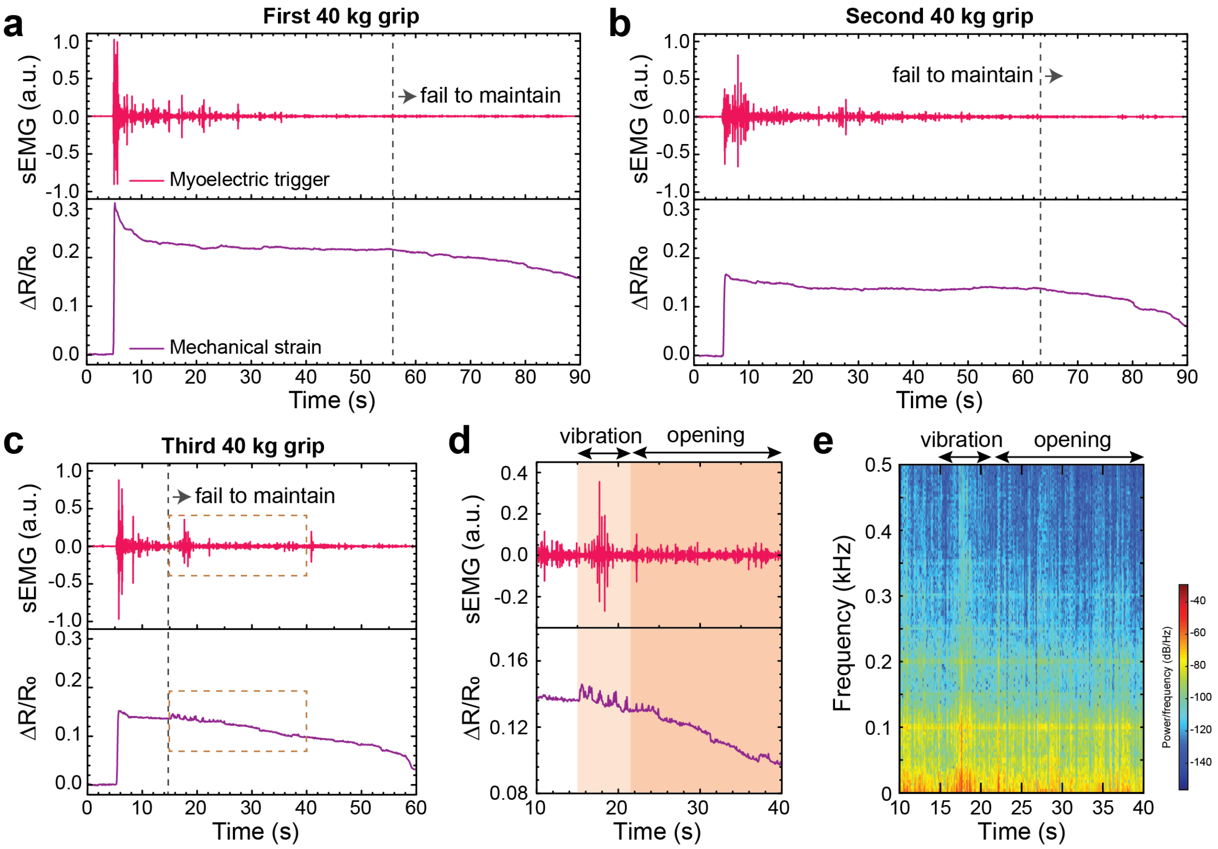

Supplementary Figure 22 | Nervous and metabolic muscle fatigue during 40 kg "grip will fail" tasks. a-c, Plots show the signatures during the three consecutive 40 kg grips with 1 min interval in between. **d,** Plot shows the window of interest in (c) that reveals the mechanical vibration initiating the gesture failure and opening of the grip, indicating the metabolic muscle fatigue. **e,** Graph shows the time-frequency analysis of sEMG signals in (d). No obvious shift was shown during the opening stage. Data obtained from subject 5, representative of two sets of consecutive grips.

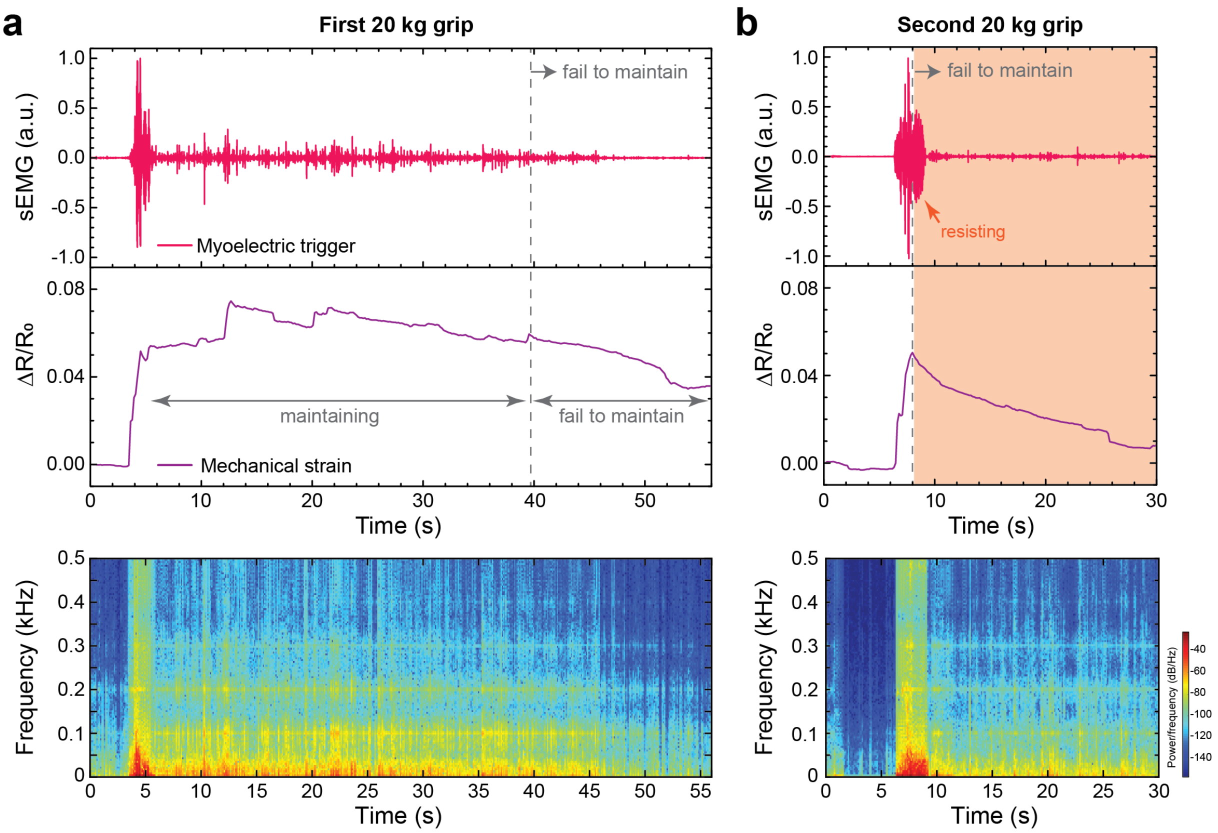

Supplementary Figure 23 | Nervous and metabolic muscle fatigue during 20 kg "grip will fail" tasks. a-b, Plots (*upper row*) show the signatures during the two consecutive grips with 1 min interval in between. Graphs (*lower row*) show the corresponding time-frequency analysis of sEMG signals. In the second grip, a relatively high level of sEMG was observed when the subject was resisting the failure of the grip gesture, whereas the drop of the mechanical strain revealed the metabolic muscle fatigue. After the resisting stage, no obvious shift in frequency or drop in the signal amplitude was observed. Data obtained from subject 2, representative of two sets of consecutive grips.

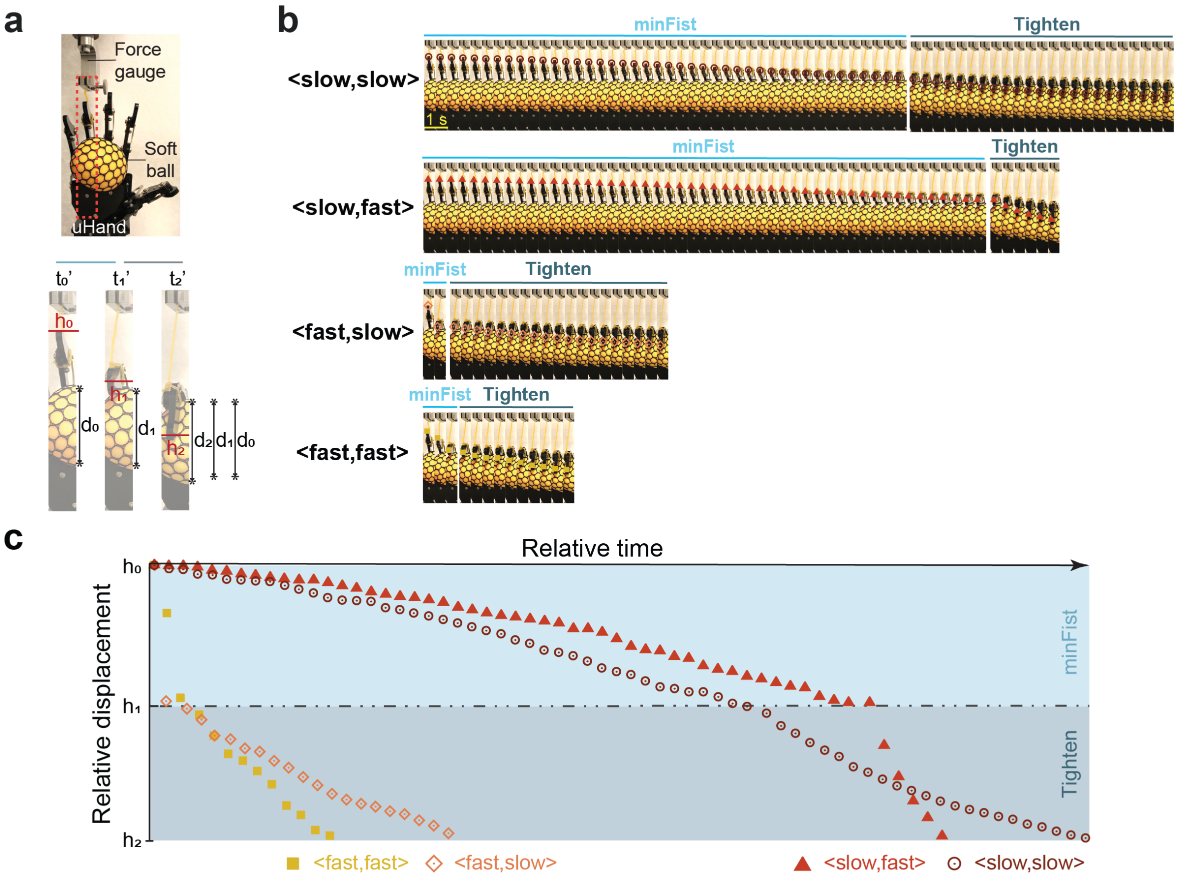

Supplementary Figure 24 | Dynamics of the uHand grips. a, Photographs showing the region of interest (*red dash rectangle*) for analyzing gesture kinetics. t_0', t_1', t_2' correspond to the time right before actuation, the end of the minFist, and the end of tightening, respectively. h_0, h_1, h_2 correspond to the position of fingertips, and d_0, d_1, d_2 indicate the projected diameter of the soft ball. d_2 is larger than d_0 and d_1, suggesting the force is only exerted on the ball during the tightening step. **b,** Kympgraphs of the region of interest in (a), with the minFist and tightening steps respectively grouped. The circle, triangle, diamond, and square symbols mark the corresponding fingertip position during respective gestures. **c,** Plot shows the dynamics analysis of the four recapitulated grip gestures in (b). Grey dash line separated the two steps of minFist and tightening.

Supplementary Table 1 | Biometric information of the subjects.

Subject	Profile	Age	Gender	Body Mass Index	Forearm Girth (cm)	Max Grip Forces (kg)
#1	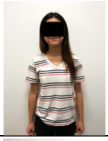	22	Female	17.5	21.1	21.0
#2	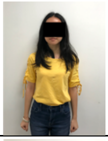	24	Female	17.6	19.5	22.5
#3	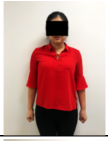	28	Female	22.7	25.2	26.4
#4	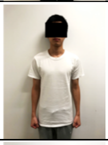	21	Male	18.0	24.9	42.0
#5	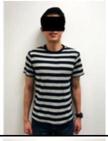	30	Male	21.0	27.5	50.6
#6	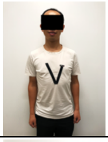	28	Male	22.0	25.9	42.1
#7	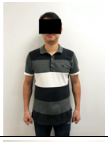	30	Male	22.0	26.0	41.5
#8	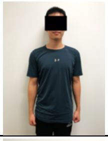	24	Male	22.5	26.5	31.1
#9	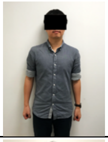	32	Male	22.5	26.5	40.8
#10	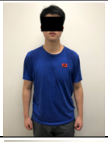	21	Male	24.1	27.1	43.6
#11	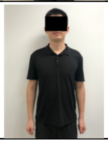	29	Male	24.7	26.4	53.1

Supplementary Note 1 | A micro model for understanding the spatiotemporal difference in correlating muscle electromechanics

A micro model at the muscle fiber level was developed by Böl *et al.*[2] in Supplementary Equation 1:

$$p^{act}(t,\lambda^{fib}) = \sum_{i=1}^{n_{FT}} \sum_{j=1}^{n_{IMP}} \rho^i \bar{F}_t^i f_t^{ij}(t) f_\lambda(\lambda^{fib}) \quad (1)$$

$\bar{F}_t$ denotes the maximal forces generated in one single muscle fiber and is correlated with the activation function $f_t(t)$, which describes the time-dependent activation level of the muscle fiber. $f_\lambda(\lambda^{fib})$ is a function of the fiber stretch λ^{fib}. Furthermore, n_{FT} denotes the number of different fiber types in the muscle, and $\rho^i = N_i^{fib}/A$ is the fiber type density, where N_i^{fib} is the number of specific fiber types and A is the cross-section area of the muscle. In addition, n_{IMP} denotes the number of impulses sent from the motor neuron to the muscle. As can be seen from the model, the electromechanical correlation of muscle contraction is highly dependent on the spatial and temporal conditions.

Supplementary Note 2 | Estimation of driving forces for delamination between *CoupOn* and skin

Fracture mechanics of a linear elastic bilayer system gives a steady-state driving force (Supplementary Equation 2) of:

$$G = \frac{1}{2} Eh\varepsilon \quad (2)$$

for interface delamination between a thin film of Young's modulus E and thickness h and a thick substrate under uniform tensile strain ε. To give a rough estimation, for the tensile/compressive strain of 80%, the driving forces for interface delamination of 40 µm-thick PDMS (E = 580 kPa) and 100 µm thick tough gel (E = 31 kPa) are 7.4 N/m and 1.0 N/m, respectively. The combined driving force (about 8.4 N/m) is much lower than the adhesion forces between hybrid *CoupOn* and skin.

Supplementary Methods:

***Synthesis of tough hydrogel*:** Tough hydrogels were synthesized following a previous study.[3] Briefly, powders of alginate (2 wt.%) and acrylamide (12 wt.%) were dissolved in deionized water. Ammonium persulphate (0.5 wt.%) as the initiator and N,N- methylenebisacrylamide (0.06 wt.% of acrylamide) as the crosslinker was then added for polyacrylamide. After briefly degassing the solution by an ultrasonic cleaner, N,N,N',N'-tetramethylethylenediamine (0.1 wt.%) was added as the crosslinking accelerator for polyacrylamide, followed by the adding of calcium carbonate nanopowders (diameter ~200 nm) as the ionic crosslinker for alginate. The gel was heated at 50 °C in a humid box overnight to cure and stabilize.

***Measurement of Young's modulus of tough hydrogel*:** Stress-strain curves were obtained by a mechanical tester (C42, MTS Systems Corporation) with controlled relative 100% humidity by an environmental chamber (Bionix Environbath, MTS Systems Corporation). The humidity of the ambient environment was measured by commercial humidity meter HI 9565 (HANNA Instruments). Tensile tests were conducted for more than three samples for each of the conditions at a speed of 1 mm/min. The Young's modulus was calculated using the linear region of the stress-strain curve.

Raman and FTIR spectroscopy analysis: TMSPMA-treated Ti/Au@elastomer was washed three times with methanol and dried with N_2 airflow, then the Raman spectrum was obtained with Confocal Raman Microscope (WITec alpha300 RS equipped with the 532 nm laser). The FTIR spectrum of three types of samples (O_2 plasma-treated Ti/Au@elastomer, TMSPMA-treated Ti/Au@elastomer, CoupOn) were obtained using FTIR spectrometer (The PerkinElmer Frontier™ IR) with Attenuated Total Reflectance (ATR) accessories from the wavenumber of 4000 cm^{-1} to 600 cm^{-1}. The *CoupOn* sample here was fabricated using 1 μL pre-gel solution and sandwiched with 200 g weight, and dried in the oven to minimize the thickness of the gel layer.

***90° peel-off tests of adhesion strength*:** To measure the interlayer adhesion between the tough hydrogel and elastomer/Au film of the hybrid *CoupOn*, the backside of elastomer was glued to a polyimide sheet (RS Components, Singapore) with superglue, and the tough hydrogel layer was bonded to TMSPMA saline-treated glass slide. The released end was gripped to a mechanical tester (C42, MTS Systems Corporation) to detect the peeling force at a speed of 10 mm/min. The adhesion strength was calculated as the force divided by the width of the

sample (1.5 cm). To measure the skin adhesion strength of hybrid *CoupOn*, the porcine skin from the supermarket was used instead of the glass after rinsing with deionized water.

Skin interfacial impedance measurement: The interfacial skin contact impedance was measured by laminating pairs of electrodes with a square shape (2 cm by 2 cm), and a center-to-center distance of 5 cm on forearm skin. As the electrodes, the *CoupOn* and commercial gel electrodes (Vitrode F150ML) were used. The interfacial impedance measurements were conducted using the electrochemical workstation (ZAHNER ZENNIUM) from 1 Hz to 1 MHz with a bias of 100 mV.

Supplementary References:

1. Z. Liu*, et al.* Highly stable and stretchable conductive films through thermal radiation-assisted metal encapsulation. *Adv. Mater.* **31,** 1901360 (2019).
2. Böl, M., Weikert, R. & Weichert, C. A coupled electromechanical model for the excitation-dependent contraction of skeletal muscle. *J. Mech. Behav. Biomed. Mater.* **4,** 1299-1310 (2011).
3. Sun, J. Y.*, et al.* Highly stretchable and tough hydrogels. *Nature* **489,** 133-136 (2012).
