# Supplementary material for: Locally coupled electromechanical interfaces based on cytoadhesion-inspired hybrids to identify muscular excitation-contraction signatures
Source: Nat Commun. 2020 May 4;11:2183. doi: 10.1038/s41467-020-15990-7 (PMC7198512; doi:10.1038/s41467-020-15990-7)
Supplement: Supplementary file 3 — Description of Additional Supplementary Files [file 41467_2020_15990_MOESM3_ESM.doc]

**Description of Supplementary Files**

**File Name: Supplementary Movie 1**

**Description:** Recognition of nervous muscle fatigue without metabolic fatigue. Subject 5 was holding a 30 kg grip for over 25s but without failure to hold it. The upper display showed the sEMG signal, and the lower display showed the muscle belly strain of FDS.

**File Name: Supplementary Movie 2**

**Description:** Robotic grip recapitulating the human grip of slow closing and slow tightening. The animated input of excitation-contraction signatures of the <slow, slow> human grip was used to command the robotic grip and generated the corresponding grip force as the output. sEMG is animated with every 10th data of the original recording.

**File Name: Supplementary Movie 3**

**Description:** Robotic grip recapitulating the human grip of slow closing and fast tightening. The animated input of excitation-contraction signatures of the <slow, fast> human grip was used to command the robotic grip and generated the corresponding grip force as the output. sEMG is animated with every 10th data of the original recording.

**File Name: Supplementary Movie 4**

**Description:** Robotic grip recapitulating the human grip of fast closing and slow tightening. The animated input of excitation-contraction signatures of the <fast, slow> human grip was used to command the robotic grip and generated the corresponding grip force as the output. sEMG is animated with every 10th data of the original recording.

**File Name: Supplementary Movie 5**

**Description:** Robotic grip recapitulating the human grip of fast closing and fast tightening. The animated input of excitation-contraction signatures of the <fast, fast> human grip was used to command the robotic grip and generated the corresponding grip force as the output. sEMG is animated with every 10th data of the original recording.
