# Supplementary material for: Locally coupled electromechanical interfaces based on cytoadhesion-inspired hybrids to identify muscular excitation-contraction signatures
Source: Nat Commun. 2020 May 4;11:2183. doi: 10.1038/s41467-020-15990-7 (PMC7198512; doi:10.1038/s41467-020-15990-7)
Supplement: Supplementary file 9 — Reporting Summary [file 41467_2020_15990_MOESM9_ESM.pdf]

nature research

Corresponding author(s): Xiaodong Chen

Last updated by author(s): Mar 9, 2020

Reporting Summary

Nature Research wishes to improve the reproducibility of the work that we publish. This form provides structure for consistency and transparency in reporting. For further information on Nature Research policies, see Authors & Referees and the Editorial Policy Checklist.

Statistics

For all statistical analyses, confirm that the following items are present in the figure legend, table legend, main text, or Methods section.

n/a	Confirmed	
☐	☒	The exact sample size (*n*) for each experimental group/condition, given as a discrete number and unit of measurement
☐	☒	A statement on whether measurements were taken from distinct samples or whether the same sample was measured repeatedly
☐	☒	The statistical test(s) used AND whether they are one- or two-sided<br>*Only common tests should be described solely by name; describe more complex techniques in the Methods section.*
☐	☒	A description of all covariates tested
☐	☒	A description of any assumptions or corrections, such as tests of normality and adjustment for multiple comparisons
☐	☒	A full description of the statistical parameters including central tendency (e.g. means) or other basic estimates (e.g. regression coefficient) AND variation (e.g. standard deviation) or associated estimates of uncertainty (e.g. confidence intervals)
☐	☒	For null hypothesis testing, the test statistic (e.g. *F*, *t*, *r*) with confidence intervals, effect sizes, degrees of freedom and *P* value noted<br>*Give P values as exact values whenever suitable.*
☒	☐	For Bayesian analysis, information on the choice of priors and Markov chain Monte Carlo settings
☒	☐	For hierarchical and complex designs, identification of the appropriate level for tests and full reporting of outcomes
☒	☐	Estimates of effect sizes (e.g. Cohen's *d*, Pearson's *r*), indicating how they were calculated

Our web collection on statistics for biologists contains articles on many of the points above.

Software and code

Policy information about availability of computer code

Data collection	Data collection listed in Methods. All software used in this study for data collection are either commercially available (Keithly 4200, Control FOUR 4.1, Spike Recorder, etc.)
Data analysis	All software used in this study for data analysis are either commercially available (Matlab 2018a, MS Excel 2016, OriginPro 9.1). Customized codes are available from the corresponding author upon reasonable request.

For manuscripts utilizing custom algorithms or software that are central to the research but not yet described in published literature, software must be made available to editors/reviewers. We strongly encourage code deposition in a community repository (e.g. GitHub). See the Nature Research guidelines for submitting code & software for further information.

Data

Policy information about availability of data

All manuscripts must include a data availability statement. This statement should provide the following information, where applicable:

- Accession codes, unique identifiers, or web links for publicly available datasets
- A list of figures that have associated raw data
- A description of any restrictions on data availability

Data that support the findings of this study are available from the corresponding author upon reasonable request.

Field-specific reporting

Please select the one below that is the best fit for your research. If you are not sure, read the appropriate sections before making your selection.

☒ Life sciences ☐ Behavioural & social sciences ☐ Ecological, evolutionary & environmental sciences

Life sciences study design

All studies must disclose on these points even when the disclosure is negative.

Sample size	Sample size was chosen according to the convenience of collecting the data and ensure the sufficient statistical power in the meanwhile.
Data exclusions	Data exclusion was considered when there was obvious electromechanical contamination or other artifacts during the tests with sEMG recordings.
Replication	Reproducibility was verified by using multiple samples or repeating identical tasks for multiple times (for each subject). All attempts at replication were successful.
Randomization	For the off-subject tests, samples were randomly chosen and tested from different batches. For subjects, subjects were randomly chosen with different age and gender, as well as varying maximum grip forces and forearm girth.
Blinding	Blinding was not relevant to my research as the participants were not grouped.

Reporting for specific materials, systems and methods

We require information from authors about some types of materials, experimental systems and methods used in many studies. Here, indicate whether each material, system or method listed is relevant to your study. If you are not sure if a list item applies to your research, read the appropriate section before selecting a response.

Materials & experimental systems

n/a	Involved in the study	
☒	☐	Antibodies
☒	☐	Eukaryotic cell lines
☒	☐	Palaeontology
☒	☐	Animals and other organisms
☐	☒	Human research participants
☒	☐	Clinical data

Methods

n/a	Involved in the study	
☒	☐	ChIP-seq
☒	☐	Flow cytometry
☒	☐	MRI-based neuroimaging

Human research participants

Policy information about studies involving human research participants

Population characteristics	11 subjects (3 female and 8 male), aged 21-32 years, with the body mass index of 17.5-24.7, the maximal forearm girth of 19.5 to 27.5 cm, the maximal grip force of 21.0 to 53.1 kg (measured with an electronic hand dynamometer Camry, EH101). No neuromuscular disorders were reported by the subjects.
Recruitment	A recruitment within the school was taken and 11 volunteers responded and participated in this study.
Ethics oversight	NTU Institutional Review Board reviewed and approved this study protocol, under Singapore's Human Biomedical Research Act (HBRA).

Note that full information on the approval of the study protocol must also be provided in the manuscript.
